# Impact of Five Cleansing Agents on Microleakage in Class V Restorations: A Comparative In Vitro Study

**DOI:** 10.1155/ijod/1323100

**Published:** 2025-07-19

**Authors:** Amir Shayegan, Candice Abergel, Clara Descombels, Nihad Malikzade, Ann-Sophie Van Hamme

**Affiliations:** Pediatric Dentistry Department, Children's Hospital of Queen Fabiola, Free University of Brussels (ULB), Brussels, Belgium

## Abstract

**Introduction:** Microleakage, caused by the failure of restorative materials to bond effectively to dental hard tissues, is a major concern in restorative dentistry. Proper treatment of dental tissues before adhesive procedures is a critical step in ensuring successful bonding. The retention of dental resin materials can be significantly improved by pretreating enamel surfaces with specific inorganic acids or chelators. This study aims to evaluate and compare the effects of five different cleanser agents on the microleakage of resin composite and glass ionomer (GI) class V restorative materials applied to enamel, dentin, and cementum.

**Materials and Methods:** Seventy-two extracted, noncarious third molars had Class V cavities prepared on the buccal and lingual surfaces and were divided into six groups: Control, ethylenediaminetetraacetic acid (EDTA)/benzalkonium chloride, EDTA + sodium fluoride, chlorhexidine, sodium hypochlorite, and aloe vera. Cleansing agents were applied for 1 min, then rinsed and gently air-dried before restoration. Buccal cavities were restored with resin composite, and lingual cavities were restored with GI cement. The obtained data were collected and statistically analyzed, with two specimens per group examined for bond quality by scanning electron microscopy (SEM).

**Results:** The results showed a significant difference in microleakage between the occlusal and cervical margins (*p* < 0.001). At the occlusal margin, the NaOCl-treated enamel group (enamel GI glass; EGIC) had the least microleakage (*p* < 0.05), while the EDTA group had the highest values.

**Conclusion:** Based on the results reported in this study, none of the cavity disinfectants in the experimental groups completely prevented microleakage. All specimens showed greater microleakage along the cervical margin than along the occlusal margin. Among the disinfectants tested, sodium hypochlorite had the lowest microleakage levels.

## 1. Introduction

In restorative dentistry, the primary goal is to restore damaged teeth' anatomical form and function. The long-term success of restoration depends on several factors, including the ability of the material to adapt precisely to the cavity and adhere effectively to the cavity walls. A persistent concern in this field is the potential for incomplete bonding to enamel and dentin, which can result in microleakage and ultimately compromise the integrity of the restoration [1].

Adequate preparation of dental tissues prior to adhesive procedures is essential to achieve durable bonding. Pretreatment of enamel with specific inorganic acids or chelating agents significantly improves the retention of resin-based materials. However, bonding to dentin poses more challenges due to its organic composition and intrinsic moisture, which can buffer the action of etching agents and reduce their effectiveness. Additionally, the surface of the cavity is typically covered with a smear layer [2], a layer of debris created during cavity preparation using rotary or hand instruments [3].

This amorphous smear layer can harbor microorganisms and inhibit optimal cavity disinfection and adhesive penetration. Its removal is, therefore, considered a key step in enhancing the success of restorative procedures [4, 5]. Acidic agents used in dentistry play multiple roles: They eliminate the smear layer and superficial dentin, expose dentinal tubules, demineralize the dentin surface, and increase the microporosity of the intertubular dentin, thereby promoting micromechanical retention.

Phosphoric acid, first introduced by Buonocore, has been widely adopted as an etchant for enamel and dentin due to its strong demineralizing and antimicrobial properties [6]. Although highly effective in removing the smear layer, strong acids can also lead to the loss of peritubular dentin, raising concerns about possible pulp irritation [7, 8]. In contrast, chelating agents such as ethylenediaminetetraacetic acid (EDTA), often combined with antimicrobial agents like benzalkonium chloride, produce a more controlled etching effect. These milder agents have been shown to enhance resin–dentin bond strength, while minimizing structural damage to the tooth substrate [9].

Chlorhexidine digluconate is widely used as an antimicrobial/antiseptic agent in plaque control and cavity disinfection. It inhibits matrix metalloproteinases (MMPs), helping reduce the levels of *Streptococcus mutans* on carious tooth surfaces and preserving dentin bond strength [10].

NaOCl is commonly used in dental procedures due to its proteolytic and disinfectant properties. It alters cellular metabolism, destroys phospholipids, and irreversibly inactivates bacterial enzymes [11–14]. Aloe vera, known for its medicinal properties, has been shown to have anti-inflammatory, antitumor, and antibacterial properties [15]. It has a matrix metalloproteinase inhibitory effect and has been used in dentistry for endodontic infections and periodontitis [16–18].

This study assessed and compared the microleakage of resin composite and glass ionomer (GI) Class V restorative materials after applying five different cleanser agents on enamel, dentin, and cementum. The null hypothesis is that different cleanser agents will not affect the bonding quality between restorations and dental hard tissue.

## 2. Materials and Methods

The study protocol for using extracted teeth was reviewed and approved by the Ethics Committee of the Children's Hospital of Queen Fabiola, Free University of Brussels (approval number CEH No. 62/15). Before collecting samples, written informed consent was obtained from all participants or their legal guardians. Seventy-two freshly extracted human third molars, noncarious, and without visible cracks, were collected. Until cavity preparation, the teeth were stored in a physiological saline solution to preserve tissue integrity. The distribution of treatment groups is detailed in Table 1.

### 2.1. Cavity Preparation

The authors adhered to a previously described protocol for cavity preparation and scanning electron microscopy (SEM) analysis [19].

After initial mechanical debridement using a hand scaler, the tooth surfaces were cleaned with a rubber polishing cup and a pumice slurry to remove residual debris. Standardized Class V cavities were then prepared on the buccal and lingual surfaces of all 72 teeth. Each cavity measured approximately 4 mm in width, 2 mm in height, and 2 mm in depth. To prevent thermal damage, preparations were carried out using a straight diamond bur (Intensiv ISO 012 FG 8714/6) under continuous air-water cooling. The coronal margins of the cavities were positioned 1 mm within the enamel, while the cervical margins extended 1 mm into the cementum. Following cavity preparation, the samples were randomly allocated into six experimental groups, with 12 teeth per group (*n* = 12).

Group A: The specimens were not treated with any cavity disinfectant and served as controls.

Group B: The cavities were pretreated with EDTA/Benzalkonium chloride (Tubulicid Blue, Dental Therapeutics AB, Saltsjö-boo Sweden) for 1 min, rinsed thoroughly with water, and dried with a mild oil-free air stream for 10 s.

Group C: The cavities were pretreated with EDTA/Benzalkonium chloride + sodium fluoride (Tubulicid Red, Dental Therapeutics AB, Saltsjö-boo Sweden) for 1 min, rinsed thoroughly with water, and dried with a mild oil-free air stream for 10 s. Tubulicid Red was reapplied to distribute fluorine.

Group D: The cavities were pretreated with a chlorhexidine digluconate 0.2% solution (Corsodyl, GlaxoSmithKline Healthcare S.A., Wavre, Belgium) for 1 min, rinsed thoroughly with water, and dried with a mild oil-free air stream for 10 s.

Group E: The cavities were pretreated with sodium hypochlorite 6% for 1 min and were rinsed thoroughly with water and dried with a mild oil-free air stream for 10 s.

Group F: The cavities were pretreated with pure aloe vera vital nonheated juice (Pur aloé, Mane en Provence, France) for 1 min and were rinsed thoroughly with water and dried with a mild oil-free air stream. After the pretreatment procedure, the Class V cavities on buccal and lingual surfaces were restored as follows.

#### 2.1.1. Buccal Cavity

After cavity preparation, enamel, dentin, and cementum surfaces were etched using 35% phosphoric acid (Ultra-Etch, Ultradent, UT, USA) and applied for 20 s on enamel and 10 s on dentin and cementum. The cavities were then rinsed thoroughly and gently dried with an oil-free air stream. Following the manufacturer's protocol, a total-etch adhesive system (Optibond FL, Kerr, USA) was applied to all cavity walls. The primer was left in place for 20 s, then lightly air-dried to eliminate excess. Subsequently, the adhesive resin was applied evenly and thinned using a gentle air stream to create a uniform layer.

Polymerization was performed using an Elipar S10 LED curing unit (3M ESPE; St. Paul, MN, USA) for 20 s. The cavity was then restored with a nanohybrid composite resin (Filtek Supreme XTE, 3M ESPE, Germany) placed in two layers; each light-cured for 40 s. Final finishing and polishing were completed using the Sof-Lex system (3M ESPE, St. Paul, MN, USA).

#### 2.1.2. Lingual Cavity

The cavity was conditioned using a 10% polyacrylic acid solution (Dentin Conditioner, GC Corporation, Tokyo, Japan) with a microbrush applicator. After conditioning the tooth surface for 20 s, the cavity was gently dried with a mild, oil-free air stream. GI resin (G.C. Fuji II LC Improved, GC Corporation, Tokyo, Japan) was then inserted in two increments, with each increment being light-cured for 20 s. Following restoration, the surfaces were coated with G.C. Fuji varnish (GC Corporation, Tokyo, Japan) for sealing. All specimens were then stored in distilled water at 37 °C and subjected to 1000 thermocycles between 5 and 55 °C, with a dwell time of 30 s in each bath and a transfer interval of 10 s.

To evaluate microleakage, 60 specimens (10 per experimental group) were coated with two layers of nail varnish, carefully leaving a 1 mm uncoated margin around the restoration site. The coated teeth were submerged in a 2% methylene blue solution for 24 h. After exposure, the samples were rinsed under running tap water to eliminate any superficial dye residues.

**Figure 1:**
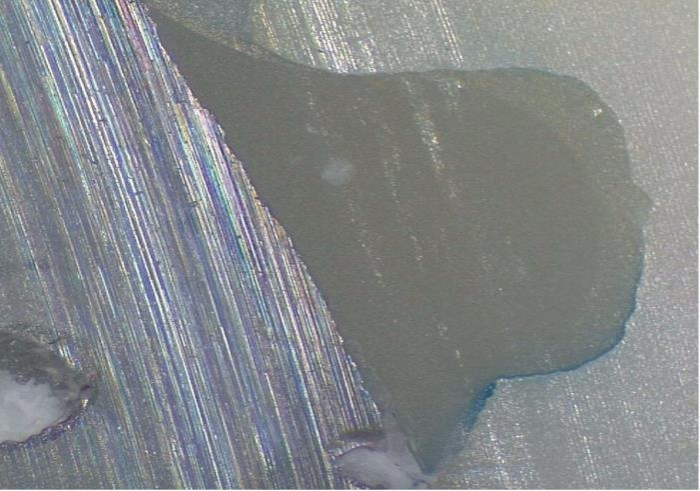
The dye penetration score is 0 in the occlusal margin and score 3 in the cervical margin. Resin composite, aloe vera group. 20 × magnification.

**Figure 2:**
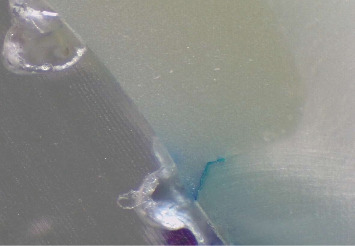
The dye penetration score is 1 in the cervical margin. Resin composite, NaOCl group. 30 × magnification.

Each tooth was then longitudinally sectioned in a bucco-lingual direction using a water-cooled diamond blade (Leitz 1600, Ernst GmbH, Wetzlar, Germany), resulting in 4–5 slices per specimen. The sections were examined using a stereomicroscope (Leica EZ4W, Leica Microsystems CMS GmbH, Wetzlar, Germany). Dye penetration was scored based on the following criteria (Figures 1 and 2):

• Score 0: No dye penetration detected.
• Score 1: Dye infiltration limited to less than half the depth of the occlusal or gingival wall.
• Score 2: Dye infiltration extending beyond half the cavity depth without reaching the axial wall.
• Score 3: Complete wall penetration up to the full cavity depth, not involving the axial wall.

All data were subjected to statistical evaluation using one-way analysis of variance (ANOVA), followed by Tukey's post hoc test for multiple group comparisons. Additionally, a Student's *t*-test was used where applicable. A *p*-value less than 0.05 was considered statistically significant. The analyses were conducted using GraphPad Prism software, version 7 (GraphPad Software Inc., La Jolla, CA, USA).

### 2.2. SEM Analysis

**Figure 3:**
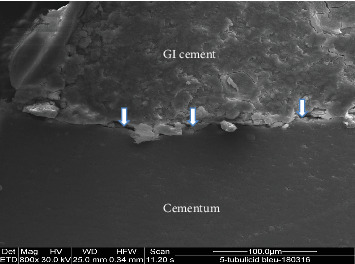
The cervical margin in the EDTA/Benzalkonium chloride group shows microleakage between glass ionomer cement (GI cement) and cementum (arrows). SEM × 800.

**Figure 4:**
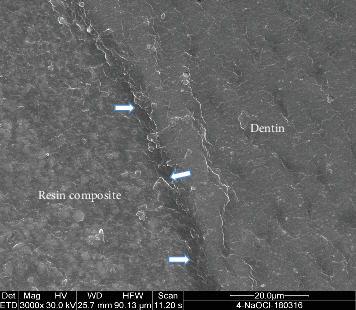
The occlusal margin in the NaOCl group shows a generalized hybrid layer formation between resin composite and dentin interfaces (arrows). SEM × 3000.

**Figure 5:**
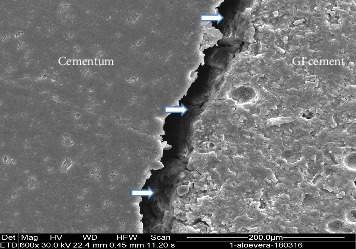
The cervical margin in the aloe vera group. Microleakage between resin composite and cementum (arrows). SEM × 600.

**Figure 6:**
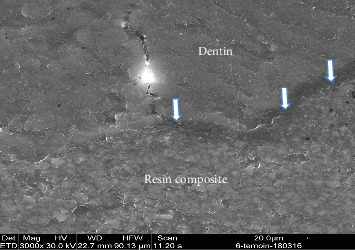
Occlusal margin in the control group. A generalized hybrid layer formation between resin composite/dentin interfaces (arrows). SEM × 3000.

Two representative specimens from each restorative subgroup (resin composite and GI cement) within each experimental group were selected for SEM analysis. The teeth were longitudinally sectioned in the bucco-lingual direction, and then dehydrated by immersion in acetone for 2 min. Subsequently, the specimens were mounted on aluminum stubs, placed under vacuum drying, and sputter-coated with a 20 µm platinum layer. Imaging was performed using a Quanta 200 SEM (FEI Company, Hillsboro, OR, USA). High-resolution micrographs were captured to evaluate the bonding interface between the restorative materials and the surrounding dental hard tissues (Figures 3–6).

## 3. Results

### 3.1. Cavity Preparation

The data collected are shown in Tables 2 and 3. There was a significant difference between occlusal and cervical margin groups (*p*  < 0.001).

#### 3.1.1. Occlusal Margin

**Figure 7:**
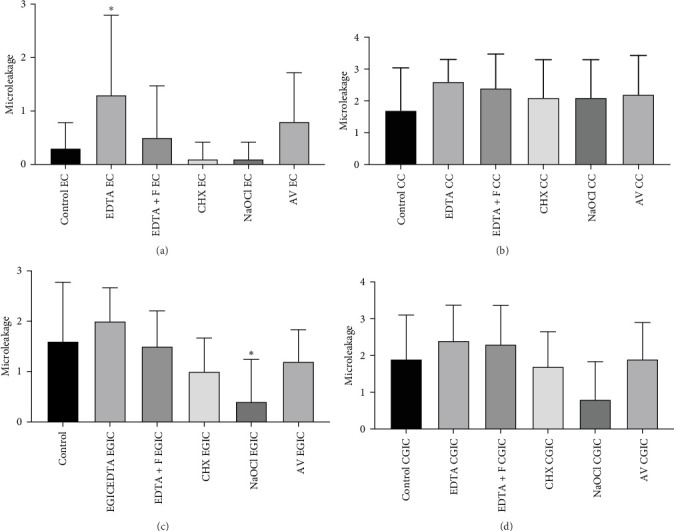
The occlusal and the cervical margin microleakage between different groups. (A) Buccal cavities: Occlusal margin microleakage between different groups. Note that there was a significant difference between groups. The EDTA group presented significantly higher microleakage values compared to the other groups (*p* =0.0333). (B) Lingual cavities: Cervical margin microleakage between different groups. Note that there was no significant difference between groups. Nevertheless, the control group presented fewer microleakage values. (C) Buccal cavities: Occlusal margin microleakage between different groups. Note that there was a significant difference between groups. The NaOCl group presented significantly lower microleakage values compared to the other group (*p* =0.0333). (D) Lingual cavities: There was no significant difference between groups. Nevertheless, the NaOCl group presented lower microleakage values.

There was a significant difference (*p*  < 0.05) between the NaOCl enamel GI glass (EGIC) group and the other groups regarding microleakage in enamel. There was no significant difference between groups. EDTA group presented more microleakage values (Figure 7A,B).

#### 3.1.2. Cervical Margin

No significant differences were observed between the groups in the cementum region. However, the NaOCl-treated CGIC group exhibited lower microleakage scores (Figure 7C,D).

## 4. Discussion

Bacteria in the smear layer significantly contribute to secondary caries, as they can remain viable for long periods. A cavity disinfectant during cavity preparation can help reduce or eliminate residual bacteria before placing the restorative material.

This study used resin composite and GI cement as adhesive materials to evaluate microleakage in Class V cavities. The cavities' cervical (apical) margins were located in cementum, while the occlusal margins were in enamel. The increased microleakage observed at the cervical margins for composite restorations may be attributed to the reduced capacity for hybrid layer formation on cementum and the inherent technical difficulties associated with bonding to dentin compared to enamel [20]. Furthermore, the absence of dentinal tubules and the higher organic content characteristic of cervical dentin likely contribute to greater leakage [21].

Phosphoric acid remains the standard agent for enhancing the adhesion between enamel and resin composites [22]. Nevertheless, forming a consistent and durable bond with dentin is more challenging due to its complex structure, which includes surface moisture and an intricate collagen matrix [23].

Enamel, dentin, and cementum samples were all collected from the same tooth to ensure consistency in chemical composition and mineralization levels, allowing for a more accurate comparison between the restorative materials.

According to the results, the NaOCl group had the lowest microleakage values, followed by the chlorhexidine group.

The impact of NaOCl on resin bond strength and microleakage is well documented. Some studies have reported that NaOCl negatively affects the hybrid layer, reducing bond strength and increasing microleakage [24, 25]. However, other studies have found no significant effect on bond strength [26, 27].

Our study found that NaOCl pretreatment resulted in the lowest microleakage at both the occlusal and cervical margins for the resin composite and GI cement groups. However, the impact of NaOCl pretreatment on composite resin's bond strength and microleakage may differ depending on the adhesive system used [28].

Therefore, it is essential to consider the adhesive system, the dentin's wettability, and the resin's ability to penetrate the substrate. Dentin bonding systems are susceptible to technique and substrate preparation.

Several studies have shown that applying 2% chlorhexidine, either before or after acid etching, does not negatively impact enamel or dentin regarding shear bond strength and microleakage when used with resin composite or GI resin [29–33].

Our study's results align with these findings. The preservation of the bond interface with chlorhexidine may be attributed to its inhibitory effect on MMPs in etched dentin. MMPs have been shown to contribute to the degradation of unprotected collagen fibrils within the hybrid layer. Therefore, MMP inhibitors such as chlorhexidine may help improve the resin bond's longevity to dentin [34, 35].

Several studies have reported higher bond strengths of resin composite to dentin when etch-and-rinse adhesive systems, rather than self-etch systems, were used after Chlorhexidine pretreatment [36, 37].

EDTA/Benzalkonium chloride-based solutions are used as cavity disinfectants in clinical practice due to their antimicrobial action and wettability properties [29, 30]. Tubulicid Red contains EDTA, benzalkonium chloride, and sodium fluoride, while Tubulicid Blue does not contain sodium fluoride. EDTA removes inorganic debris as a smear layer, and benzalkonium chloride has three essential functions: An antimicrobial agent, a cationic surfactant, and a matrix metalloprotease inhibitor.

In our study, Tubulicid Red and Blue showed higher microleakage values along the occlusal and cervical margins in the resin composite and GI cement groups. It has been suggested that Tubulicid may interfere with the bonding of etch-and-rinse adhesives because its components, benzalkonium chloride and sodium fluoride, may be incorporated into the residual smear layer, making it more resistant to phosphoric acid etching [31].

There is a global need for an alternate cavity disinfectant that is safe and effective. With the growing popularity of natural products, they are considered better alternatives to synthetic chemicals [38]. Therefore, we used naturally available aloe vera for cavity disinfection before restorative treatment in the present study. *Aloe vera* (Aloe barbadensis miller) contains aloins, barbadoins, and anthraquinones responsible for antimicrobial activity and is a potent inhibitor of MMPs-2 and 9 [15, 18]. The MMP inhibitory activity of aloe vera is attributed to thealoins, which are potent inhibitors of stimulated granulocyte MMPs. The results of previous studies showed that aloe vera has potent bactericidal activity against both cariogenic and periodontopathic bacteria [16, 39].

Several studies have evaluated the effect of aloe vera on the bond strength of composite resin to dentin and shown that applying aloe vera to acid-etched dentin improves the longevity of the resin–dentin bond. To our knowledge, few studies have been conducted on the effects of aloe vera on GI bond strength to dental hard tissue. Our study showed that aloe vera pretreatment did not significantly affect the microleakage values compared to the control group.

## 5. Conclusion

Within the limitations of this in vitro study, none of the cavity disinfectants in the experimental groups completely prevented microleakage. All specimens showed greater microleakage along the cervical margin than the occlusal margin. Among the disinfectants tested, sodium hypochlorite had the lowest microleakage levels when used with the restorative techniques and materials in this Class V study on permanent teeth. Further long-term in vivo studies are recommended to investigate this issue.

## Figures and Tables

**Table 1:** Distribution of treated teeth.

Group A *n* = 12	Group B *n* = 12	Group C *n* = 12	Group D *n* = 12	Group E *n* = 12	Group F *n* = 12
Control	EDTA	EDTA + sodium fluoride	Chlorhexidine digluconate 0.2%	Sodium hypochlorite 6%	Aloe vera

**Table 2:** Grading of dye penetration of occlusal margin.

Control EC	Control EGIC	EDTA EC	EDTA EGIC	EDTA + F EC	EDTA + F EGIC	CHX EC	CHX EGIC	NaOCl EC	NaOCl*⁣*^*∗*^ EGIC	AV EC	AV EGIC
1	0	0	1	0	1	0	1	0	0	0	1
0	3	0	2	1	2	0	0	0	0	0	1
0	1	3	1	0	1	0	1	0	0	0	2
0	1	3	2	0	1	0	1	0	2	2	1
1	3	3	3	0	1	0	0	0	0	1	1
0	2	3	3	1	1	0	1	0	0	1	1
0	2	1	2	3	2	0	1	0	2	0	0
0	1	0	2	0	2	0	1	1	0	0	1
0	3	0	2	0	1	1	2	0	0	2	2
1	0	0	2	0	3	0	2	0	0	2	2

Abbreviations: AV, aleo vera; CHX, chlorhexidine; EC, enamel composite; EGIC, enamel glass ionomer glass.

*⁣* ^*∗*^ Significant difference (*p* < 0.05).

**Table 3:** Grading of dye penetration of cervical margin.

Control CC	Control CGIC	EDTA CC	EDTA CGIC	EDTA + F CC	EDTA + F CGIC	CHX CC	CHX CGIC	NaOCl CC	NaOCl CGIC	AV CC	AV CGIC
3	2	3	3	3	3	3	2	3	1	3	1
1	3	3	0	1	2	2	1	0	0	2	2
2	3	3	3	3	3	3	0	2	0	3	2
3	2	2	3	3	3	3	2	3	2	3	3
0	1	3	3	3	3	2	3	3	3	2	2
0	0	2	2	3	1	0	2	2	0	0	3
2	3	3	2	0	3	0	3	2	1	0	3
0	3	3	3	2	0	2	2	0	0	3	0
3	2	1	2	3	3	3	1	3	1	3	1
3	0	3	3	3	2	3	1	3	0	3	2

Abbreviations: AV, aleo vera; CC, cement composite; CGIC, cement glass ionomer glass; CHX, chlorhexidine.

## Data Availability

The data that support the findings of this study are available from the corresponding author upon reasonable request.
